# OSAS in children: clinical and polysomnographic respiratory profile

**DOI:** 10.1016/S1808-8694(15)30968-X

**Published:** 2015-10-19

**Authors:** Regina Terse Trindade Ramos, Carla Hilário da Cunha Daltro, Paloma Baiardi Gregório, Leda Solano de Freitas Souza, Nilvano Alves de Andrade, Antônio de Souza Andrade Filho, Almerio de Souza Machado Júnior

**Affiliations:** a MD, MS; b MD, MS in Internal Medicine by the Federal University of Bahia; c Specialist in Sleep Medicine; d PhD in Internal Medicine – Federal University of Bahia; e PhD in Medicine – University of São Paulo Medical School; f PhD in Medicine – Federal University of Rio de Janeiro; g MD, MS in Internal Medicine. Postgraduate Course in Medicine and Health – Bahia School of Medicine and Public Health Fundação Bahiana para Desenvolvimento das Ciências/Fundação Oswaldo Cruz; Hospital Português

**Keywords:** sleep apnea, obstructive sleep apnea, child, polysomnography, snoring, sleep

## Abstract

Obstructive sleep apnea and hypopnea syndrome in children (osas) has an estimated prevalence of up to 3% and can be associated with neurocognitive and behavioural abnormalities, and also cardiovascular complications. This study may help pediatricians, who are unaware of the problem, to recognize osas.

**Study design:**

series of cases.

**Aim:**

to describe the clinical characteristics and polysomnographic respiratory findings in a population of children with obstructive sleep apnea and hypopnea syndrome referred to the sleep laboratory from january 2002 up to july 2003.

**Methods:**

we studied 93 patients between 2 and 10 years of age with polysomnographic diagnosis of obstructive sleep apnea and hypopnea syndrome. Age, gender, racial group and questions about the children's health and sleep related disorders were evaluated. Apneahypopnea index, oxyhemoglobin desaturation, and arousal index were evaluated too.

**Results:**

males represented 61.3%, With a mean age of 5.2 ± 2.1 (Years-old). The complaints that most commonly lead to the exams were snoring in 24.7% And restless sleep in 24.7%. Associated medical conditions frequently reported were allergic rhinitis (98.9%) And adenoid hypertrophy (50.6%). Mild apnea was found in 66%. The mean and sd of spo2 nadir was 89.1 ± 3.5% And the mean and sd of the number of arousals was 8.4 ± 3.5/Hour of sleep. Conclusion: the results suggest the possibility that obstructive sleep apnea and hypopnea syndrome should be suspected in children with allergic diseases and adenoid and tonsil hypertrophy with snoring and restless sleep complaints.

## INTRODUCTION

The first report of sleep-related respiratory disorders in children was made in 1836, when Charles Dickens in his book: “The Posthumous Papers of the Pickwick Club”^1^, described a 10 year old boy who spent most of his time eating and sleeping, similar description of some current reports of patients with Obstructive Sleep Hypopnea/Apnea Syndrome (OSHAS). Sir William Osler^2^, in his medical textbook from 1892, described childhood OSHAS, discussing daily symptoms and the extremely disturbed sleep patterns of these children.

In 1976, Guilleminault et al.^3^ described a series of eight children with sleep apnea, diagnosed by polysomnography. Five years later, Guilleminault et al.^4^ published a review paper describing 50 children and teenagers with obstructive sleep apnea, concluding that the syndrome was not so rare and its cardiovascular and intellectual impact should be considered.

Childhood OSHAS is characterized by recurring episodes of upper airway complete and/or partial obstruction during sleep, resulting in intermittent hypoxemia and hypercapnia, frequent awakings and sleep pattern breaks^5^, ^6^. It is a severe condition in children and differs from its adult counterpart in its physiology, clinical presentation, polysomnographic characteristics and sequels. The estimated prevalence is of 1 to 3% in children; however, it is difficult to measure it, because of subdiagnosis^6^, ^7^, ^8^, ^9^, ^10^. Lack of community awareness about the negative sleep-related effects on children daily functioning together with parents underestimation of the problem when they talk to the physician are factors that contribute to this underestimation^7^.

The most prevalent age group is in pre-school age children, between 3 and 5 years - time of the greatest lymphoid tissue growth, and also in adolescence^7^, ^8^. Its etiology is multifactorial and is specially associated to adenotonsillar hypertrophy^8^, ^9^. There is no Brazilian data about OSHAS prevalence in children.

The most common complaints are snoring and difficulty to breath during sleep; some children may present difficult and noisy breathing^11^. Obesity is a risk factor for OSHAS, however most of the children with this problem are not obese^18^. A great number of apneic children, when awake show completely normal physical exam, and this also contributes to diagnosis delay^17^.

The gold standard test for OSHAS diagnosis is the night time polysomnography; because the patient's clinical history alone is not enough to establish a definitive diagnosis^9^.

Treatment advocated for most children with OSHAS and adenotonsillar hypertrophy, without other diseases, is adenotonsillectomy. Notwithstanding, many of the operated children may have symptom recurrence during adolescence^11^. When surgery fails, we usually indicate Upper Airway Continuous Positive Pressure therapy (CPAP)^12^. Long term childhood prognosis is unknown^5^.

This study proposed to assess the clinical findings and polysomnographic respiratory indices, documented by polysomnography, in OSHAS children studied in a sleep lab. The goal is to contribute to facilitate the OSHAS diagnosis for pediatricians and revise indication criteria for polysomnographic studies based on clinical data.

## MATERIALS AND METHODS

The present paper is a descriptive study, case series with retrospective polysomnographic data collection. Our sample was non-probabilistic and made up of 93 children with OSHAS, between two and ten years of age, who underwent polysomnographic exam at the Hospital Português Sleep Lab, seen between January 2002 and July 2003. Exclusion criteria included: children below two years and above 10 years of age; genetic disease, cerebral palsy, neuromuscular diseases or any other systemic disease. Following the identification of those children that matched the inclusion criteria, the parents were asked to participate in the study. If they consented, they were asked to fill out a detailed questionnaire prepared by the authors, based on questionnaires presented in the literature^13^, ^14^. In this questionnaire, demographic and medical data were assessed, as well as details about problems related to the child's sleep patterns in the last year. Among many other issues, the questionnaire asked if the child snored during sleep, if the sleep was restless, if there were respiratory pauses, behavioral problems or problems with school learning. Behavioral problems were considered present if a positive answer were obtained for any of the following questions: “Has anyone ever considered your child aggressive?” Learning problems were considered if a positive answer were given to the following question: “Does your child's teacher think your child has learning disorders at school?” Presence of asthma, rhinitis, adenoid hypertrophy, tonsils hypertrophy was considered as positive answers to the questions: “In the last year was your child diagnosed with asthma? Rhinitis? Adenoid Hypertrophy? Tonsils hypertrophy?

The children were classified in two age-ranges: children with five years or less, and those children above five years of age, because at five years we see the peak growth of lymphoid tissue^6^, ^10^.

In a nutritional assessment, growth parameters were obtained through standardized growth graphs from the National Center for Health Statistics. They were expressed according to the sleep lab experience where the research was carried out, using the modified Waterlow criterion, based on the height/age (H/A) and weight/height indexes (W/H). Children whom the adequacy percentage were ³120%, were considered as classical obese. Eutrophic between ³ 91% and < 110%; and malnourished were those < 91%^15^, ^16^.

The standardized night time polysomnographic assessment was carried out at the sleep lab, using a 16 channel Respironics (Healthdyne Alice 4system) computerized device. The children were studied for a minimum period of 5 hours and maximum of 10 hours in a sound treated room, with light and temperature control, accompanied by one of the parents, without previous sedation or sleep deprivation. The reports were issued and revised by a sleep medicine specialist physician. The following tests were recorded: electroencephalogram, electro-oculogram and electromyogram (electrode on the chin and on one of the legs); and the following parameters were measured: movement of both the chest and abdominal wall, heart rate through a electromyogram and oronasal air flow through a nasal thermistor (Thermistor Airflow Sensor, 6210). Oxygen arterial saturation (SpO2) was assessed by means of a pulse oxymeter (Healthdyne Technologies Oximeter). Sleep architecture was assessed by the standard technique and the amount of time spent in each sleep stage was expressed as a percentage of the total sleep time. Central, obstructive and mixed-type apneas were recorded. Respiratory events were thus defined: central apnea was defined as a lack of oronasal airflow – measured by nasal thermistor without respiratory effort; central apnea with duration equal or greater than 10 seconds was quantified. Obstructive apnea was defined as the presence of chest and abdominal wall movements in the absence of oronasal airflow measured by the thermistor, of 5 seconds duration or longer. Hypopneas were defined as a 50% or more reduction in nasal airflow associated to the paradoxical movement of the chest and a 4% or more reduction in oxyhemoglobin saturation. The apneas with both components – central and obstructive – were classified as mixed apneas and included in the apnea/hypopnea index (AHI). The AHI was calculated through the total sum of obstructive hypopneas and apneas, plus the mixed apneas, divided by the total time of sleep^17^, ^18^. Apnea and hypopnea values equal or above 1 were considered abnormal. Those children with AHI values between 1 and 5 events/hour of sleep were considered as having light OSHAS; moderate OSHAS were those with AHI between 5.1 and 10; and the severe cases were those with AHI ³ 10.1 event/hour of sleep^18^.

Arterial oxygen saturation values below 92% and 10 or more micro-awakenings/hour of sleep were considered abnormal^11^, ^19^.

In order to build the data bank and make the statistical calculations, we used the Statistical Package for the Social Sciences software. The continuous variables were expressed as average ± standard deviation, added to the median if the variable distribution were not normal. The category variables were expressed as proportions. We used the t Student test to compare the variables between the two groups for independent samples or the Mann-Whitney, according to the distribution of the variable considered. In order to compare proportions we used the Pearson Chi-squared or Exact Fisher test. The present study was submitted to and approved by the Ethics committee of Research with Human Beings of the Portuguese Hospital. The children guardians signed the informed consent for their inclusion in the study.

## RESULTS

Table 1 depicts the demographics of the children with polysomnographic diagnosis of OSHAS.

**Table 1:** Demographics of 93 OSHAS children from January 2002 to July 2003.

Characteristics	Values
Gender	

Males	57 (61,3%)
Females	36 (38,7%)
Age in complete years	
Average and standard deviation	5,2 ± 2,1
Median	5,0

Racial group	

White	14 (15%)
Brown	76 (82%)
Black	3 (3%)

Adjusted weight/height	

Average and standard deviation	107 ± 16,4
Median	105

The adjusted Weight/Height variable varied from 73% to 166% with average of 107.0 ± 16.4%. 7.5% of the children were malnourished, while 62.4% were eutrophic, 12.9% were slightly overweight and 17.2% were obese. The average of the Weight/Height adjusted variable in both male and female children were, respectively, 108.5 ± 15.7% and 106.3 ± 17.5%, without statistical difference when both groups were compared (p=0.473). Assessing the children according to weight (children with normal weight and overweight children) and the degree of apnea, there were no statistical difference between the groups (p=0.462).

**Chart 1:**
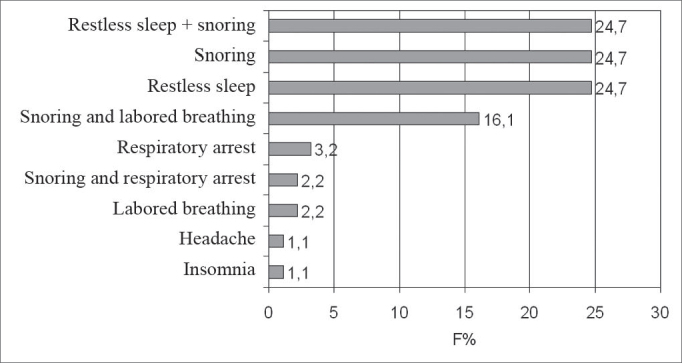
Frequence of the complaints that led to a polysomnographic exam in 93 OSHAS children, in a sleep lab, from January 2002 to July 2003.

Table 2 depicts the general characteristics of the children with OSHAS referred to the sleep lab, divided by gender; and Chart 1 shows the frequence of complaints that led to the requirement of a polysomnography.

**Table 2:** Distribution, according to age in complete years, Weight/Height ratio (W/H), Hypopnea and Apnea Index (HAI) and rate of micro awakenings per hour of sleep for children with polysomnographic diagnosis of OSHAS, according to gender, from January 2002 to July 2003.

	Girls n= 36 (38.7%)	Boys n= 57 (61.3%)	p Value
Age (years) *	4,6 ± 1,9	5,6 ± 2,2	0,041
W/H (adjusted) +	106,3 ± 17,5	108,5 ± 15,7	0,473
HAI (events/hour)*	3,7 ± 2,6	5,6 ± 6,0	0,067
Micro awakenings/hour*	7,1 ± 4,0	8,5 ± 4,3	0,144

* Mann-Whitney test

+ t Student test

The polysomnography was first indicated by pneumologists in 81.7% of the cases studied, by pediatricians in 11.8%, by otorhinolaryngologists in 3.2% and by neurologists in 3.2% of the children.

**Chart 2:**
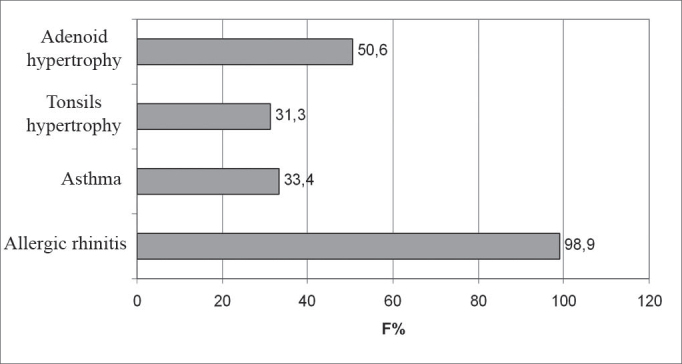
Frequence of medical conditions related to these 93 OSHAS children, in a sleep lab, from January 2002 to July 2003.

According to the parents’ response to the questionnaire, 93.5% of the children snored at night; 88.2% had restless sleep and 54.8% had difficulty breathing during sleep; 93.5% of the children had nasal obstruction during sleep and 64.5% were daily restless. Chart 2 shows de frequence of medical conditions associated to these children.

Of the 93 children with OSHAS, nine had undergone surgical removal of lymphoid tissues, two underwent adenoid and tonsil removal and seven underwent adenoid removal alone.

The HAI varied from 1 to 34.6 events/hour of sleep, with average of 4.9 ± 5.1 (median: 3.6) events/hour of sleep. As to the apnea severity, 69.9% of the children had light apnea, 22.6% had moderate episodes and 7.5/% were diagnosed as having severe apnea.

Minimum oxygen saturation varied between 79% and 97%, with average of 89.1 ± 3.6%. As to the number of micro arousals/hour, there was a variation of 0.1 to 47 micro arousals/hour, with an average of 8.4 ± 5.9 (median: 8.0) micro arousals/hour of sleep.

The patients were also analyzed in two age groups, using 5 years as a cutting point. 53.6% of the children up to 5 years of age were males and in those patients above 5 years, 73% were males. In the groups with less and more than 5 years, the proportion of individuals of both genders did not vary significantly (p=0.06). As to nutritional status variation, we observed that in the group of children aged below or equal to 5 years, 7.1% were malnourished, 60.7% eutrophic, 16.1% overweight and 16.1% were obese; with the children older than 5 years of age, 8.1% were malnourished; 64.9% eutrophic, 8.1% were overweight and 18.9% were truly obese. The average of the Weight/Height adjusted variable found in children of 5 years or younger was of 106.7 ± 14.3%; in the group of children above 5 years of age, the average found for this variable was of 109.1 ± 19.2%, (p=0.482), both are values found in eutrophic children.

Table 3 depicts the general characteristics of children with OSHAS, according to age group. Light OSHAS was present in 66.1% of the children aged 5 years or less, and in 74.7% in those older than 5 years of age (p=0.509).

**Table 3:** General traits of OSHAS children, divided by age groups, from January 2002 to July 2003.

Gender *			
Males	30 (53,6%)	27 (73%)	p=0,060
Females	26 (46,4%)	10 (27%)	
Adjusted W/H +			
Average and standard deviation	106,7 ± 14,3	109,1 ± 19,2	p=0,482
Snoring *	55 (98,2%)	32 (86,5%)	p=0,024
Labored breathing during sleep *	33 (58,9%)	18 (48,6%)	p=0,330
Feeling of suffocation *	24 (42,9%)	8 (21,6%)	p=0,035
Nasal obstruction*	53 (94,6%)	34 (91,9%)	p=0,679
Daily restlessness *	39 (69,6%)	21 (56,8%)	p=0,204
Aggressiveness *	2 (3,6%)	2 (5,4%)	p=1,000
Good school performance *	47 (83,9%)	20 (54,1%)	p = 0,002
Parents snore *	29 (51,8%)	15 (40,5%)	p=0,288
Parents smoke *	2 (3,6%)	3 (8,1%)	p=1,000

* Chi-Squared test

+ t Student test

According to gender, the boys aged 5 or less presented higher hypopnea and apnea indexes when compared to girls [6.7 ± 7.2 (median: 4.5) events/hour of sleep vs. 3.6 ± 2.7 (median: 2.6) events/hour of sleep; p=0.018]. In those children above 5 years of age, average HAI for girls was of 4.0 ± 2.4 (median: 2.7) events/hour of sleep, and in males it was of 4.4 ± 4.2 (median: 3.2) events/hour of sleep (p=0.837). The average of the minimum oxygen saturation in children aged 5 years was of 88.8 ± 3.5% and in those children older than 5 years of age, the average was of 89.4 ± 3.6% (p=0.419).

## DISCUSSION

The present study describes the clinical characteristics and the polysomnographyc respiratory indices of 93 children with OSHAS, clinically stable, who underwent polysomnography in a sleep lab in the city of Salvador.

We observed that 61.3% of the apneic episodes happened more to boys than to girls of the population studied. In adults it has been shown that one of the strong factors related to OSHAS predisposition is being male^10^. Redline et al.^20^ showed that in children, the gender did not influence much on the risk of developing sleep-related respiratory disorders, thus suggesting that the differences seen in adults may be hormone-mediated, having its influences in respiratory control and the distribution of body fat, and they would probably play a discreet role in pre-adolescent children^21^.

Racial distribution analysis revealed that 85% of the population were brown and black. This is due to the fact that most of the people who live in Bahia State are brown or black. About 70% of the State population is made up of brown and black people, and this ratio is even higher in the city of Salvador (77.5%)^22^. Many publications reveal a greater prevalence of OSHAS in black children, and this is probably due to cranio-facial characteristics of African descendants^20^.

The disorder prevailed in pre-school children, similar to data already published in the literature^5^, ^6^, ^21^.

OSHAS children seem to have their growth impaired^21^. Most children in the present study were eutrophic and only 7.5% of the children were malnourished. Different ideas have been proposed to explain the low weight gain in OSHAS children, such as IGF-I depression during sleep and also the low caloric intake of patients with adenotonsillar hypertrophy^23^. 17.2% of the children assessed were obese. Obesity has been related to a 4 to 5 fold increase in the risk of developing OSHAS in children, when compared to their non-obese counterparts^20^.

Polysomnographies were mainly ordered by pneumologists and only 11.8% of the requests were made by pediatricians. Children with OSHAS may be seen by many specialists. Usually pediatricians are more visited because of indirect complaints of low weight/height development, and pneumologists because of snoring and labored breathing^24^. Often times, parents downplay their children's sleep problems to pediatricians in medical consultations. Stein et al.^25^ assessed 472 children aged between 4 to 12 years, and the parents of 10.8% of these children reported persistent problems related to sleep in the last six months; notwithstanding, less than 50% of these children's parents talked about their children's sleep during the medical consult. Smedje et al.^26^ have shown that, although sleep problems were common in a population of 1844 children between 5 and 7 years of age, this issue was raised in the medical consults by the parents in only 6.7% of the cases. The reasons why the parents downplayed the sleep problems seem to be multifactorial. It is possible that this may have been influenced by memory biases, as well as by the limited knowledge the parents have in relation to the importance of potential OSHAS sequels in children, such as learning disabilities, disorders of the neurocognitive function and daily behavior. It is important to remember that the pediatrician, as he/she records the patient's history, may also inhibit or downplay the parent's complaints about their children sleep disorders. There is evidence that pediatricians may underreport sleep problems in children^27^. It is possible that sleep-related problems are discussed during the medical visit; however they are not documented, nor duly appreciated. These findings, altogether, point to significant gaps in understanding sleep problems in clinical practice.

Clinical manifestations found in this study are in agreement with literature data. The complaints that motivated the polysomnographic studies were mainly restless sleep, snoring, breathing breaks during sleep and labored breathing^4^, ^21^, ^23^. It was seen that breathing difficulty during sleep was an important symptom indicative of OSHAS, being more significant in children younger than 5 years of age.

Snoring was reported for most of the children. OSHAS primary symptom is snoring and its presence indicate an increase in upper airway resistance. We noticed that children below five years of age snored more than their older counterparts. At this age, the adenoid tissue is still relatively large in relation to the size of the airway. In school aged children and in teenagers, the nasopharynx increases in size, while the lymphoid tissue remains stable or reduces in size, thus creating a larger airway^28^.

Although the report of restlessness was subjectively evaluated in the questionnaire, most children were considered restless during the day, by their parents. Hyperactivity, aggressiveness and lack of attention, as well as memory impairment and learning disability are found in children with OSHAS; after being submitted to adenotonsillectomy, they improved, thus suggesting that neurocognitive deficits are, at least, partially reversible^7^, ^29^.

OSHAS was strongly associated to the presence of nasal obstruction and allergic rhinitis, referred by the parents in the population studied. Allergic rhinitis, commonly found in children, is an important cause of nasal obstruction. Nasal influence in snoring and obstructive sleep apnea is well known. 30 Physiopathologically, rhinitis causes nasal mucosa edema and mucous secretion that increase nasal resistance, thus predisposing the child to complete or partial obstruction of upper airways during sleep. It is possible that the increase in nasal airway resistance generates an increase in negative inspiratory pressure, thus causing a turbulence of lax soft tissue and a collapse of upper airway causing obstruction and OSHAS^31^. McColley et al.^30^ showed that children with allergic rhinitis had more diagnosis of OSHAS when compared to controls, although they did not see differences as far as OSHAS severity is concerned.

Even having polysomnography as a common procedure in children, reference values are yet to be standardized in the different pediatric sleep labs of the world. Due to the large amount of data obtained from the polysomnographic study, we chose to use three extremely important parameters only in this study: HAI, oxyhemoglobin saturation during sleep and the number of micro awakenings/hour of sleep. We also observed that the average of HAI corresponded to mild apnea and the indexes are much lower than those seen in adults with OSHAS, which reference values used to brand someone as apneic are defined as of 5 events per hour of sleep^9^.

OSHAS severity interpretation requires the consideration of other respiratory parameters, such as oxyhemoglobin arterial saturation levels; the observed average of oxyhemoglobin saturation in children during sleep was of 92.3 ± 2.4%, within the range considered as normal values by Marcus et al.^18^. The average of desaturation observed in our children was compatible to low intensity desaturation. As to the number of micro awakenings per hour of sleep, its average was similar to that seen in children with no respiratory disorder related to sleep in other pediatric sleep labs^11^, ^21^.

The importance of this study is that its findings are easily generalized to other sleep labs in their daily practices. Patients here enrolled probably represented the children along the entire OSHAS presentation spectrum. One limitation is that it only assessed children with documented abnormality found in polysomnography, and this did not allow us to compare these children with the population without polysomnographic alterations compatible with OSHAS. The lack of a control group precluded the definition about the importance of each OSHAS-related finding. How frequent physicians from different specialties ordered polysomnography was certainly influenced by the greater advertisement of sleep labs made to pneumologists.

## CONCLUSION

Most of the children analyzed in this study have mild intensity clinical signs, complaints of snoring, restless sleep and the feeling of suffocation during sleep, specially those below 5 years of age. Daily restlessness was frequently reported by the parents. Most of the children had nasal obstruction, and allergic rhinitis was the most frequently mentioned disease, followed up close by adenotonsillar hypertrophy. Most polysomnographic exams in this series were ordered by a pneumologist.
